# Predicting Undernutrition at Age 2 Years with Early Attained Weight and Length Compared with Weight and Length Velocity

**DOI:** 10.1016/j.jpeds.2016.11.013

**Published:** 2017-03

**Authors:** Catherine Schwinger, Lars T. Fadnes, Sanjaya K. Shrestha, Prakash Sundar Shrestha, Ram Krishna Chandyo, Binob Shrestha, Manjeswori Ulak, Ladaporn Bodhidatta, Carl Mason, Tor A. Strand

**Affiliations:** 1 Centre for International Health, Department of Global Public Health and Primary Care, University of Bergen, Bergen, Norway; 2 Department of Clinical Dentistry, University of Bergen, Bergen, Norway; 3 Research Unit, Walter Reed/Armed Forces Research Institute of Medical Sciences, Kathmandu, Nepal; 4 Department of Child Health, Institute of Medicine, Tribuhvan University, Kathmandu, Nepal; 5 Department of Enteric Diseases, Armed Forces Research Institute of Medical Sciences (AFRIMS), Bangkok, Thailand; 6 Department of Research, Innlandet Hospital Trust, Lillehammer, Norway

**Keywords:** Anthropometry, WHO Growth Velocity Standards, early identification, malnutrition, stunting, wasting, underweight, AUROC, Areas under the receiver operating characteristic curves, LAZ, Length-for-age z score, LVZ, Length velocity z score, MAL-ED, The Etiology, Risk Factors, and Interactions of Enteric Infections and Malnutrition and the Consequences for Child Health and Development study, WAZ, Weight-for-age z score, WHO, World Health Organization, WLZ, Weight-for-length z score, WVZ, Weight velocity z score

## Abstract

**Objective:**

To estimate the abilities of weight and length velocities vs attained growth measures to predict stunting, wasting, and underweight at age 2 years.

**Study design:**

We analyzed data from a community-based cohort study (The Etiology, Risk Factors, and Interactions of Enteric Infections and Malnutrition and the Consequences for Child Health and Development study [MAL-ED] study) in Bhaktapur, Nepal. A total of 240 randomly selected children were enrolled at birth and followed up monthly up to age 24 months. Linear and logistic regression models were used to predict malnutrition at 2 years of age with growth velocity z scores at 0-3, 0-6, 3-6, 6-9, 6-12, and 9-12 months (using the World Health Organization Growth Standards) or attained growth at 0, 3, 6, and 12 months as predictors.

**Results:**

At age 2 years, 4% of the children were wasted, 13% underweight, and 21% stunted. Children who were malnourished at age 2 years had lower mean growth z scores already at birth and throughout the study period. Anthropometric indicators in infancy were significant predictors for growth at the age of 2 years during most periods and at most ages in infancy. Weight-for-age z score, length-for-age z score, and weight-for-length z score at age 12 months had excellent areas under the curve (91-95) to predict the value of the same indicator at age 24 months. Maximum area under the curve values for weight and length velocity were somewhat lower (70-84).

**Conclusions:**

Growth measured at one time point in infancy was better correlated with undernutrition at age 2 years than growth velocity.

The first 1000 days of life, starting from conception until around the child's second birthday, increasingly are recognized as essential for child growth, with inadequate growth often indicating serious and potentially irreversible consequences.^1^, ^2^, ^3^, ^4^, ^5^ Childhood undernutrition is estimated to contribute to 45% of all the deaths of children younger than 5 years globally^6^; however, early anthropometric deficits also are associated with long-term consequences for health and educational attainment, extending into adulthood and even into the next generation.^3^, ^4^, ^7^, ^8^, ^9^ Thus, the first 1000 days have been suggested to be critical for the prevention of malnutrition.

Any measure of inadequate attained growth used for identifying children at risk of adverse events has the inherent limitation that the child already is stunted or wasted to a varying degree, impeding possibilities for prevention and impacts of nutritional interventions. Longitudinal growth measures such as weight velocity or weight gain have a theoretical advantage as they present a picture of the current growth trend, whereas attained growth is a cumulative measure of an altered growth rate that leads to a recognizable malnourished state.^10^, ^11^ Few studies have estimated the extent to which measures of longitudinal growth early in life can predict future nutritional status. Although weight at 12 months predicted stunting at 36 months equally well as weight gain from 3 to 6 months in children living the Republic of Congo,^12^ the detection at an earlier age with weight gain could be advantageous. Iannotti et al^13^ found that weight gain during the first month of life predicted attained weight and length at 1 year of age, but they did not compare it with attained growth measures. In a study in Peru, no advantage of weight gain assessment to predict underweight at 24 months of age was found compared with attained weight assessment.^11^ Length gain was not found predictive of wasting or stunting at later ages in Peru and Guatemala.^11^, ^14^ These studies all had different approaches to define weight and length gain.

The World Health Organization (WHO) published growth velocity standards in 2009,^15^ offering the opportunity to score weight and length gain according to age and sex. Two studies have used the WHO growth velocity standards to assess the relationship with future nutritional status, but one focused on the association with obesity and did not compare the predictive ability of weight velocity with other growth measures,^16^ and the other studied children with cystic fibrosis in the US.^17^ Studying growth velocities could help to identify critical time windows for prevention or early interventions of undernutrition.^7^, ^8^, ^18^ We therefore aimed to estimate the abilities of weight and length velocity z scores in infancy (according to the WHO Child Growth Standards) to predict stunting, wasting, and underweight at the age of 2 years and compare them with those of the attained growth measures weight-for-age z score (WAZ), length-for-age z score (LAZ), and weight-for-length z score (WLZ).

## Methods

The Etiology, Risk Factors, and Interactions of Enteric Infections and Malnutrition and the Consequences for Child Health and Development study (MAL-ED) was conducted in 8 countries (Bangladesh, Brazil, India, Nepal, Pakistan, Peru, South Africa, and Tanzania). For this analysis, data from the Nepal site were used. The study in Nepal was carried out in the Bhaktapur municipality, located 15 km east of the capital Kathmandu and at about 1400 m above sea level. Bhaktapur had a population of about 78 000 people in 2010.^19^ Hinduism and Buddhism are the predominant religions practiced in this municipality, and community members are primarily distinguished by the traditional caste system. Tourism and agriculture are the main sources of livelihoods. The climate is humid subtropical, with a hot and wet monsoon season from May to September and a cool and dry season from October to March. A pilot study in 2010 of 100 households with children 24-36 months of age showed that although socioeconomic indicators compared favorably with national averages, 40% of children were stunted.^19^

The MAL-ED study is a prospective cohort study. During enrollment from June 2010 to February 2012, 668 deliveries were recorded, with 97% occurring at the hospital. Deliveries outside the hospital were registered by fieldworkers surveying the households. Households with recent deliveries were selected randomly on a weekly basis. The number for children selected each week was based on a prestudy census, which informed the expected birth rate, and the target sample size defined for all 8 sites of the MAL-ED study (ie, to arrive at >200 children enrolled during a period of 2 years).^20^

With this weekly number, 275 children were selected, and all caretakers of were informed about the MAL-ED study. If informed consent was given, households were screened for enrollment. Participants were excluded if the family had plans to move out of the catchment area for >30 consecutive days during the first 6 months of follow-up; the mother was <16 years of age; the mother had another child already enrolled in the MAL-ED study; the child was not a singleton (ie, twins, triplets); the child's guardian failed to provide signed informed consent; weight at birth or enrollment was <1500 g; or the infant had any of the following indications of serious disease: hospitalization for something other than a typical healthy birth; severe or chronic condition diagnosed by a medical doctor (eg, neonatal disorder; renal, liver, lung, and/or heart disease; congenital conditions); or enteropathies diagnosed by a medical doctor. In total, 240 children were enrolled. Ethical approval for the study was obtained from the Nepal Health Research Council and the Walter Reed Institute of Research (Silver Spring, Maryland). All caretakers of the participating children provided informed consent. This subanalysis was approved by the Central Board of the MAL-ED study.

At enrollment (within 17 days after delivery), well-trained fieldworkers interviewed caretakers on the child's date of birth, birth weight (available for 97% of the children), breastfeeding status, and sociodemographic characteristics of the household and took anthropometric measurements using standardized techniques (length, weight, and head circumference). Thereafter, monthly anthropometric measurements were taken until the age of 2 years, resulting in 24 anthropometric measurements for each child. Length was measured with a standard length board (ShorrBoard; Weigh and Measure, LLC, Olney, Maryland), weight with an infant scale (seca, Chino, California), and head circumference with a nonstretch synthetic tape (seca). Each month a supervisor duplicated 10% of the measurements within 24 hours. The interobserver technical error of measurement for these repeated measurements was 0.343 for height and 0.070 for weight.

### Data Management and Statistical Analyses

If concern or suspicion was articulated during measurements, raw values were plotted on growth curves. In case of implausible discrepancies to the previous values, measurements were redone immediately. All data were double-entered into a local database, and discrepancies and completeness were checked by the site data entry supervisor. If necessary, remeasurements were taken within the shortest time possible, generally within 2 days. Data were sent to and stored at the Data Coordinating Center at Fogarty International Center (Bethesda, Maryland), which did an external quality control and marked values that exceeded plausible ranges within subsequent measurements (increments >1.5 kg for weight, >3.5 cm for length, and >2 cm for head circumference) for review by the study site. The Data Coordinating Center made Web-based issue logs available to the local teams to enable prompt corrections. In addition, monthly reports provided the sites with feedback on data quality.

Data were analyzed with Stata (version 13; StataCorp LP, College Station, Texas). We calculated WAZ, WLZ, LAZ, weight velocity z score (WVZ), and length velocity z score (LVZ) according to the WHO Child Growth Standards.^15^, ^21^ We defined wasting, stunting, and underweight as z score ≤−2 for WLZ, LAZ, and WAZ, respectively.

For the description of the sample, we report percentages, means with SDs or medians with IQRs as appropriate. For each anthropometric index, we built a separate simple logistic or linear regression model, depending on the format of the outcome, ie, WAZ, WLZ, or LAZ at 2 years of age as continuous variable (linear) or as dichotomous variable with a cut-off at −2 z scores (logistic). The predictor variables, all tested in the regression models one at a time, were the individual growth velocity z scores, for 3- and 6-month increments at the ages 0-3, 0-6, 3-6, 6-9, 6-12, and 9-12 months as well as measures of attained growth at the ages 0, 3, 6, and 12 months. Because weight at birth was lacking for 7 children (3%), we imputed values for birth weight for those by regressing birth weight from the earliest weight measurements. Length was not measured at birth and therefore length within 17 days was used as proxy for birth-length for all 240 children. For all other target ages, we allowed for a deviation of ±3 days, eg, between 2.9 and 3.1 months at the 3-month visit.

For linear regression models, the R-square is reported in addition to the regression coefficients. For logistic regression models, receiver operating characteristic curves depict the balance between sensitivity and specificity at different threshold levels. ORs and areas under the receiver operating characteristic curves (AUROC) are reported.

## Results

In total, 240 children were enrolled into the study, of which 130 (54%) were male and 233 (97%) delivered at a health facility. Characteristics of the study sample are summarized in Table I. The majority of the mothers (90%) initiated breastfeeding within the first 24 hours after childbirth. Introduction of solid foods was on average at 3 months, although supplementary liquids were given earlier. On an average, exclusive breastfeeding lasted 1 month (IQR 0.6-3.2 months) and total breastfeeding duration 24 months (IQR 23-26 months). A toilet with a flush to a piped sewer system was available in 94% of the households, although 46% of those shared facilities with up to 10 other households. The median monthly household income was approximately 12 000 Nepali rupees (IQR 8000-20 000), corresponding to about 144 US$ (IQR 95-240).

**Figure 1:**
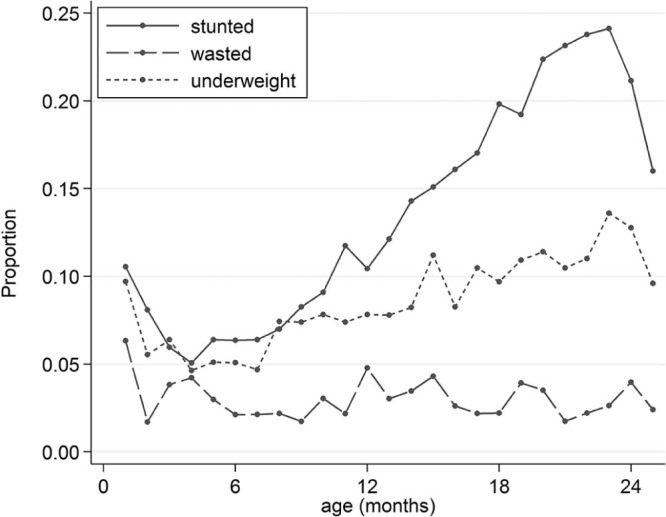
Proportion of 240 children enrolled in the MAL-ED study, Nepal, being stunted, wasted, or underweight according to age. Stunting is defined as LAZ < −2, wasting as WLZ < −2, and underweight as WAZ < −2, according to the WHO Child Growth Standards.

**Figure 2:**
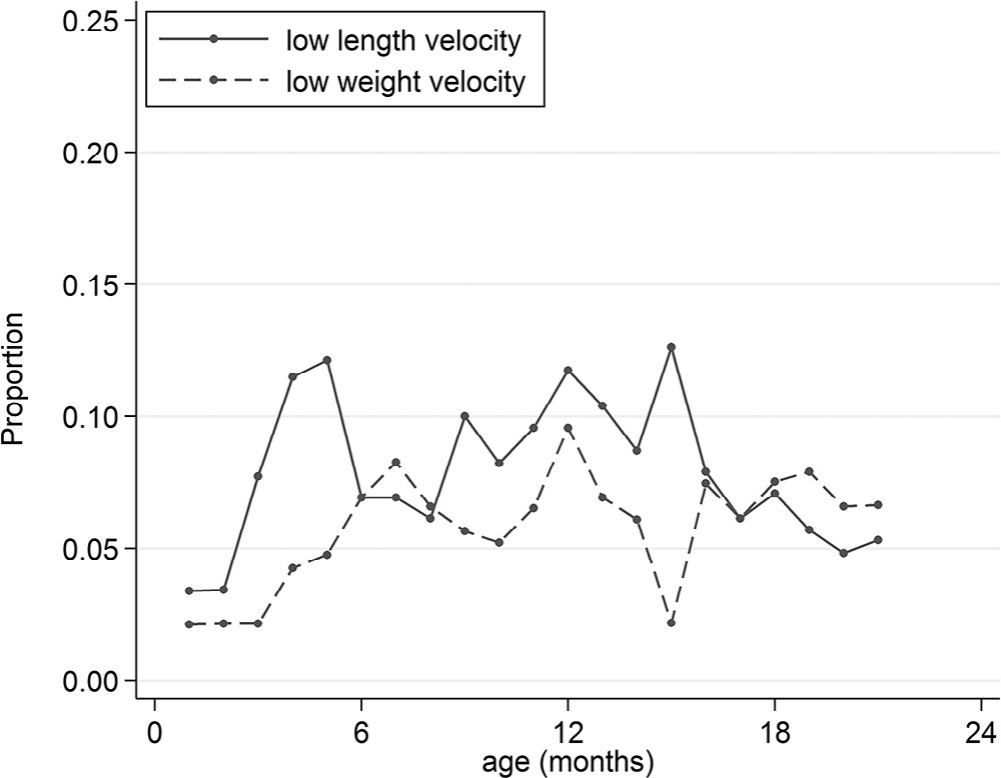
Proportion of 240 children enrolled in the MAL-ED study, Nepal, having a low weight or length velocity z score (<−2) according to age. All velocity z scores use a 3-month increment and are plotted at the beginning of the growth period, eg, a velocity z score plotted at 3 months is the velocity for the period from 3 to 6 months.

At the age of 2 years, 4% of the children were classified as wasted (WLZ ≤ −2), 13% as underweight (WAZ ≤ −2), and 21% as stunted (LAZ ≤ −2). Figure 1 displays the proportion of children who were wasted, underweight, and stunted according to age and correspondingly for low weight and length velocity z scores (≤−2) in Figure 2 (available at www.jpeds.com). Mean weight and length velocity z scores for the whole study sample were above the standard mean in the first 3 months but declined with age until about 5 and 13 months, respectively, and improved marginally thereafter. Indicators for mean attained growth (WAZ, WLZ, and LAZ) were low already at birth, improved slightly until about 5 months of age, and deteriorated continuously after that age.

**Figure 3:**
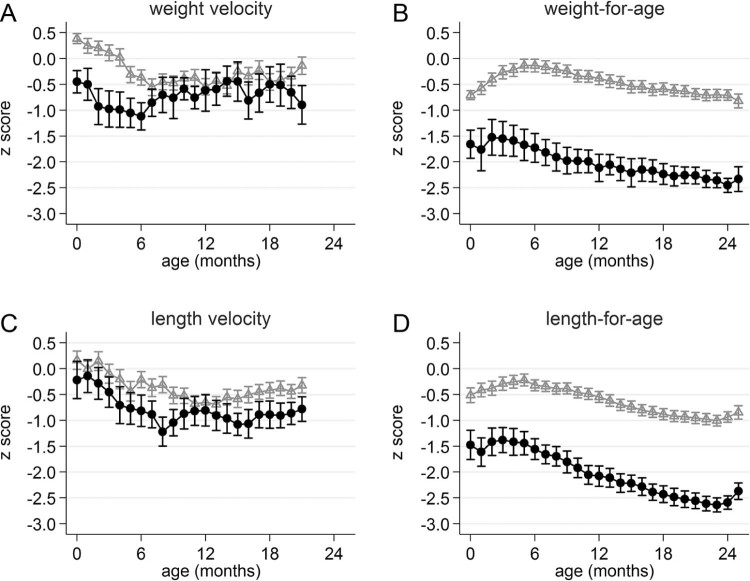
Mean z scores for different anthropometric indices with 95% CI over time from age 0 to 24 months according to **A** and **B,** underweight at 24 months or **C** and **D,** stunting at 24 months (yes = *black circles*; no = *gray triangles*) of 240 children enrolled in the MAL-ED study, Nepal. Underweight and stunting is defined as WAZ and LAZ < −2, respectively. All velocity z scores use a 3-month increment and are plotted at the beginning of the growth period, eg, a velocity z score plotted at 3 months is the velocity for the period from 3 to 6 months.

When we compared children who were underweight or stunted at 2 years of age with those who were not, it showed that differences in mean z scores for WAZ and LAZ were apparent already at birth and remained throughout the study period up to 2 years of age (Figure 3, B and D). For those underweight at 2 years of age, mean weight velocity z scores werelower for the periods starting during the first 6 months of life (Figure 3, A). For those stunted at 2 years of age, length velocity z scores were lower throughout the first 2 years of life. However, there were some periods where there was a substantial overlap of the 95% CIs of the growth velocity estimates (Figure 2, C).

Linear and logistic regression models showed that most periods of the different anthropometric indicators were significant predictors for growth at the age of 2 years. A general trend could be observed, ie, indicators of attained growth during the first year predicted attained growth at 2 years of age better than velocity z scores, Table II, Table III, Table IV (Tables III and IV available at www.jpeds.com). LAZ at 12 months could explain 75% of the variation in LAZ at 24 months, whereas LVZ from 6 to 12 months only explained 24%. For WLZ at 2 years, the R^2^ of WLZ at 6 months was 0.50 and for WVZ between 0 and 6 months 0.28. Also, more variation of WAZ at 2 years was explained by an indicator of attained growth (WAZ at 12 months, R^2^ = 0.66), than by a velocity z score (WVZ 0-6 months, R^2^ = 0.44). The value for AUROC ranged between 91 and 95 for WAZ, LAZ, and WLZ at 12 months and was somewhat lower for weight and length velocity at different time periods (70-84). No difference between girls and boys could be observed. The trend that the older the children, the better the ability to predict nutritional status at 2 years of age, as seen in indicators of attained growth, did not appear in indicators for growth velocity.

## Discussion

In this study, indicators of attained growth during the first year of life predicted stunting, wasting, and underweight at age 2 years better than velocity z scores. WAZ, LAZ, and WLZ at age 12 months had excellent AUROC (91-95) to predict the value of the same indicator at age 24 months. Maximum AUROC values for weight and length velocity in different growth periods were somewhat lower (70-84).

In agreement with our study results, Simondon et al^12^ found weight measured at one time point (12 months) to be most predictive of stunting at age 1-5 years. Nevertheless, in their study, weight velocity from 3 to 6 months had equally high sensitivity and specificity values. They used predicted quarterly weight gains as velocity measure, which does not take measurement error and transient weight losses into account. Temporary weight loss, eg, weight loss caused by disease with catch-up growth during recovery, is well described in literature. Advantages of predictions by modeled growth velocity become clear, although the disadvantage for practical settings, where only raw measurement values are used, needs to be emphasized.

In children with cystic fibrosis in the US, attained growth measures (WAZ and LAZ) at age 4 months predicted low WAZ and LAZ (<10th percentile) at 24 months better than WVZ and LVZ at different age periods^17^ when the WHO Child Growth Standards were used. The authors argue that one reason why attained growth indictors performed better might have been that growth velocity z scores were more sensitive to the therapy of cystic fibrosis. Although velocity z scores increased after the introduction of therapeutic measures, they were still insufficient for most children. They would have needed to be positive for a sufficient amount of time to counterbalance completely the growth deficit seen in attained growth measures. Similar reasons could have interfered with the predicting ability of weight and length velocity z scores in our study, because children found sick were referred to health services and treated. This explanation is strengthened by the findings of a study with children from all 8 sites of the MAL-ED study, in which rates of growth defined by a linear piecewise model were found to be greater after periods with high enteropathogen detections. Still, values for indicators of attained growth were decreasing with age.^22^

In the study of Ruel et al,^14^ anthropometric indicators were ranked in the same order for their ability to predict stunting at age 3 years as they were in our study, ie, LAZ performed best followed by WAZ, WVZ, LVZ, and WLZ. Attained growth indicators performed better in children aged 6 months compared with 3 months. For children with cystic fibrosis in the US, early attained weight and length (at 4 months) was more predictive than at later ages (6, 12, 18 months), but in their study sample, more children were classified as undernourished in early ages, because the underlying cause (cystic fibrosis) was treated. In our study, the proportion of malnourished children increased with increasing age. This continuous deterioration in nutritional status typically is seen in low-income countries,^2^ leading to better predictions of future nutritional status with increasing age as seen in our analysis.

Our hypothesis, that velocity z scores would perform better to predict future growth, was based on the theoretical idea that low growth rates would accumulate and in the end lead to a detectable low nutritional status. This was described in a study in Guatemala,^11^ where mean weight-for-age of the study sample was not below standard mean before 5 months, although weight gains already were lower much earlier. Even though growth velocity z scores during several time periods significantly predicted growth at age 2 years in our study, they performed worse than attained growth measures. In earlier analyses, however, we have shown that growth velocities were better than attained growth to predict child death within 3 months,^23^ supported by other studies where growth velocity was lower in the time period just before death whereas no association was shown with attained indices.^24^, ^25^ We also found that velocity z scores could depict changes in growth according to the well-known seasonal cycle of food availability in an area heavily depending on subsistence farming, which was not apparent in attained growth.^26^ This might point to the important advantage of growth velocities over attained growth measures of being able to capture current risk factors, thus representing the current risk profile and better predict short term health consequences.

Differences in mean z scores for weight-for-age and length-for-age already were apparent at birth, emphasizing the importance of intrauterine life and other prenatal factors for optimal growth development. Differences remained throughout the study period, with persistently lower weight and length velocity z scores in those children that were malnourished at age 2 years further augmenting the difference in attained growth. This gives an indication that infancy is still an important period to avoid or hamper critical growth deficits at later ages.

The study has several strengths, including being community-based, with random selection of the children, and only a few children lost to follow-up (5% at the end of the study), reducing the possibility of selection bias. Most children were measured within accurate 1-month intervals with a thorough validation procedure, allowing for a very strict definition of target ages (±3 days) for this analysis. Compared with this, the study by Heltshe et al^17^ allowed for ±9 days' deviation; however, length was not measured at birth, and length measurements within the first 17 days were used as proxy for birth length. Therefore, length velocity z scores in the periods 0-3 and 0-6 months are artificially low as the result of less time to grow in these 3- and 6-months periods, which could have influenced the predictive abilities in these age periods. Nevertheless, additional analyses, in which we estimated birth length from regression lines based on the 2 subsequent length values, did not change the results substantially (data not shown). Low goodness of fit of the models of LVZ also in later age periods (assessed by R^2^ and AUROC values) supports the robustness of our findings.

Our results show that measuring growth at one time point in infancy seems to be sufficient to distinguish between those at high risk of becoming malnourished and those at lower risk. For low-income settings with high prevalence of malnutrition, where resources are often scarce, simplicity of growth monitoring is likely to encourage health personnel to actually do it. The same conclusion is given by Piwoz et al,^11^ where weight gain was the best predictor for weight-for-age at 12 months, but because of the favored simplicity, the authors advised using attained weight for monitoring programs. Malnutrition, however, remains an enormous problem in low-income countries with serious consequences and efforts need to be put into optimizing detection and treatment of it. We would like to point out the value of assessing growth cross-sectionally and longitudinally, both reflecting different aspects of growth. The decision on which of the methods to use needs to be evaluated carefully, taking into account the purpose and the resources available. Despite the possible drawbacks of growth velocities concerning their practicality at present,^23^, ^27^ because their greater sensitivity to capture influencing factors,^26^ their potential to predict short-term consequences,^23^ and their strength to reflect the dynamics of growth rather than status, we think that they could be a valuable tool for research in the field of malnutrition that merits further study.

## Figures and Tables

**Table III:** Early attained growth and growth velocity z scores on WLZ or wasting (WLZ < −2) at age 24 months

	n	Linear regression		Logistic regression	
		Coefficient (95% CI)	R^2^	OR (95% CI)	AUROC
WVZ, mo					
0-3	227	0.40(0.30,0.50)	0.22	0.51(0.28,0.93)	0.70
0-6	221	0.45(0.36,0.55)	0.28	0.50(0.26,0.94)	0.69
3-6	160	0.32(0.21,0.43)	0.17	0.84(0.40,1.76)	0.59
6-9	183	0.19(0.06,0.32)	0.05	0.91(0.41,2.02)	0.57
6-12	181	0.21(0.07,0.35)	0.05	0.96(0.40,2.34)	0.52
9-12	218	0.10(−0.03,0.23)	0.01	0.61(0.32,1.15)	0.60
LVZ,mo					
0-3	186	−0.08(−0.19,0.03)	0.01	1.18(0.66,2.13)	0.57
0-6	182	−0.01(−0.13,0.11)	0.00	0.89(0.41,1.94)	0.53
3-6	160	0.07(−0.04,0.19)	0.01	1.15(0.56,2.37)	0.51
6-9	183	0.04(−0.08,0.17)	0.00	1.33(0.63,2.81)	0.59
6-12	181	0.05(−0.08,0.18)	0.00	2.03(0.83,4.95)	0.66
9-12	218	0.03(−0.09,0.14)	0.00	1.44(0.77,2.70)	0.65
WAZ,mo					
0	227	0.36(0.23,0.48)	0.12	0.36(0.18,0.73)	0.71
3	188	0.54(0.43,0.65)	0.34	0.38(0.20,0.73)	0.78
6	188	0.64(0.54,0.73)	0.49	0.28(0.12,0.66)	0.77
12	223	0.58(0.49,0.67)	0.42	0.33(0.18,0.62)	0.82
LAZ,mo					
0	227	0.17(0.06,0.29)	0.04	0.57(0.31,1.04)	0.65
3	188	0.16(0.03,0.30)	0.03	0.54(0.26,1.12)	0.63
6	188	0.26(0.11,0.41)	0.06	0.61(0.26,1.44)	0.60
12	223	0.18(0.06,0.31)	0.04	0.77(0.38,1.54)	0.53
WLZ,mo					
0	224	0.25(0.14,0.35)	0.08	0.68(0.37,1.24)	0.63
3	188	0.46(0.36,0.56)	0.31	0.47(0.25,0.89)	0.74
6	188	0.60(0.52,0.69)	0.50	0.22(0.08,0.58)	0.84
12	223	0.60(0.52,0.68)	0.49	0.26(0.12,0.55)	0.91

**Table IV:** Early attained growth and growth velocity z scores on WAZ or underweight (WAZ < −2) at age 24 months

	n	Linear regression		Logistic regression	
		Coefficient (95% CI)	R^2^	OR (95% CI)	AUROC
WVZ, mo					
0-3	227	0.47 (0.38, 0.56)	0.30	0.37 (0.24, 0.57)	0.78
0-6	221	0.55 (0.47, 0.64)	0.44	0.22 (0.13, 0.38)	0.84
3-6	160	0.38 (0.28, 0.49)	0.25	0.38 (0.22, 0.66)	0.81
6-9	183	0.28 (0.15, 0.40)	0.09	0.52 (0.34, 0.81)	0.73
6-12	181	0.38 (0.24, 0.51)	0.15	0.41 (0.24, 0.68)	0.75
9-12	218	0.18 (0.05, 0.31)	0.03	0.68 (0.45, 1.02)	0.59
LVZ, mo					
0-3	186	0.03 (−0.08, 0.14)	0.00	0.81 (0.56, 1.19)	0.57
0-6	182	0.12 (−0.00, 0.23)	0.02	0.52 (0.32, 0.84)	0.68
3-6	160	0.12 (0.01, 0.23)	0.03	0.75 (0.51, 1.11)	0.62
6-9	183	0.18 (0.06, 0.31)	0.05	0.64 (0.41, 0.98)	0.62
6-12	181	0.27 (0.15, 0.40)	0.10	0.63 (0.40, 0.98)	0.63
9-12	218	0.15 (0.03, 0.27)	0.03	0.85 (0.57, 1.26)	0.54
WAZ, mo					
0	227	0.40 (0.28, 0.52)	0.16	0.32 (0.20, 0.53)	0.76
3	188	0.59 (0.49, 0.69)	0.42	0.23 (0.13, 0.40)	0.87
6	188	0.72 (0.64, 0.80)	0.63	0.07 (0.03, 0.18)	0.93
12	223	0.72 (0.65, 0.79)	0.66	0.06 (0.02, 0.15)	0.95
LAZ, mo					
0	227	0.34 (0.23, 0.44)	0.15	0.39 (0.26, 0.59)	0.77
3	188	0.41 (0.28, 0.53)	0.19	0.20 (0.10, 0.38)	0.84
6	188	0.63 (0.51, 0.76)	0.35	0.06 (0.02, 0.17)	0.91
12	223	0.60 (0.50, 0.70)	0.39	0.15 (0.08, 0.28)	0.86
WLZ, mo					
0	224	0.11 (−0.00, 0.22)	0.02	0.68 (0.47, 0.98)	0.59
3	188	0.31 (0.21, 0.42)	0.15	0.65 (0.44, 0.98)	0.63
6	188	0.51 (0.41, 0.61)	0.35	0.37 (0.22, 0.62)	0.76
12	223	0.56 (0.47, 0.64)	0.44	0.21 (0.12, 0.37)	0.86

**Table I:** Selected characteristics of the study sample of 240 children aged 0-24 months living in Bhaktapur municipality, Nepal, enrolled into the MAL-ED study, 2010-2012

	n	Values
Male sex, %	240	54
Education, father		
Ever gone to school, %	104	95
Median duration of education, y (IQR)	99	9 (6-10)
Education, mother		
Ever gone to school, %	236	94
Median duration of education, y (IQR)	221	10 (6-10)
Median household incomes, median (IQR)*	236	12 (8-20)
Electricity available, %	236	100
Access to flush toilet, %	236	94
Owning a television, %	236	94
Owning a computer, %	236	25
Owning a refrigerator, %	236	25
WAZ, mean (SD)		
0-6 mo	2088	−0.52 (1.00)
7-12 mo	1353	−0.52 (0.99)
13-24 mo	2653	−0.82 (0.93)
LAZ, mean (SD)		
0-6 mo	1855	−0.57 (0.98)
7-12 mo	1354	−0.77 (0.93)
13-24 mo	2653	−1.20 (0.93)
WLZ, mean (SD)		
0-6 mo	1851	−0.12 (1.11)
7-12 mo	1353	−0.15 (1.01)
13-24 mo	2653	−0.33 (0.91)

* Nepali rupees per month in thousands, corresponding to about 144 US$ (IQR 95-240).

**Table II:** Early attained growth and growth velocity z scores on LAZ or stunting (LAZ < −2) at 24 months

	n	Linear regression		Logistic regression	
		Coefficient (95% CI)	R^2^	OR (95% CI)	AUROC
WVZ, mo					
0-3	227	0.33(0.23,0.44)	0.14	0.46(0.32,0.65)	0.71
0-6	221	0.42(0.32,0.52)	0.23	0.37(0.25,0.54)	0.75
3-6	160	0.28(0.17,0.39)	0.13	0.58(0.39,0.85)	0.72
6-9	183	0.26(0.13,0.39)	0.08	0.56(0.39,0.82)	0.68
6-12	181	0.41(0.28,0.55)	0.17	0.43(0.27,0.68)	0.73
9-12	218	0.20(0.06,0.33)	0.04	0.84(0.59,1.18)	0.55
LVZ,mo					
0-3	186	0.16(0.05,0.27)	0.04	0.74(0.53,1.02)	0.61
0-6	182	0.22(0.10,0.35)	0.07	0.65(0.43,0.91)	0.65
3-6	160	0.13(0.01,0.24)	0.03	0.80(0.58,1.10)	0.59
6-9	183	0.28(0.16,0.40)	0.11	0.47(0.32,0.70)	0.70
6-12	181	0.44(0.33,0.56)	0.24	0.29(0.18,0.47)	0.79
9-12	218	0.24(0.13,0.36)	0.07	0.54(0.37,0.77)	0.67
WAZ,mo					
0	227	0.27(0.14,0.41)	0.07	0.47(0.32,0.69)	0.68
3	188	0.39(0.26,0.51)	0.17	0.37(0.24,0.56)	0.74
6	188	0.48(0.37,0.59)	0.27	0.27(0.16,0.45)	0.78
12	223	0.56(0.47,0.66)	0.37	0.25(0.16,0.40)	0.81
LAZ,mo					
0	227	0.40(0.29,0.51)	0.20	0.37(0.26,0.54)	0.76
3	188	0.54(0.43,0.65)	0.32	0.22(0.13,0.39)	0.82
6	188	0.82(0.72,0.92)	0.57	0.08(0.04,0.18)	0.90
12	223	0.87(0.80,0.93)	0.75	0.02(0.01,0.07)	0.95
WLZ,mo					
0	224	−0.13(−0.24,−0.01)	0.02	1.16(0.84,1.60)	0.57
3	188	−0.02(−0.14,0.10)	0.00	0.92(0.67,1.27)	0.50
6	188	0.13(0.01,0.26)	0.02	0.73(0.51,1.04)	0.58
12	223	0.23(0.11,0.34)	0.07	0.67(0.49,0.92)	0.61
